# Reported Prevalence and Associated Factors of PTSD and Posttraumatic Stress Symptoms Among Individuals With Diabetes: A Scoping Review

**DOI:** 10.1155/jdr/6218315

**Published:** 2026-11-09

**Authors:** Zhang Yuanping, Cao Ruiqing, Ke Xiong, Jing Yuanyuan

**Affiliations:** ^1^ School of Nursing North Sichuan Medical College Nanchong China nsmc.edu.cn; ^2^ Key Laboratory of Digital-Intelligent Disease Surveillance and Health Governance North Sichuan Medical College Nanchong China nsmc.edu.cn; ^3^ School of Management North Sichuan Medical College Nanchong China

**Keywords:** associated factors, diabetes, posttraumatic stress symptoms, prevalence, PTSD

## Abstract

**Background:**

Posttraumatic stress disorder (PTSD) and posttraumatic stress symptoms (PTSS) may occur among individuals with diabetes, but reported estimates and associated factors vary considerably across studies. This scoping review is aimed at mapping the reported prevalence of PTSD/PTSS and factors associated with these outcomes among individuals with diabetes.

**Methods:**

A scoping review was conducted in accordance with the PRISMA‐ScR guidelines. Web of Science, PubMed, Embase, Cochrane Library, CNKI, Wan Fang Data, and VIP were systematically searched for empirical studies reporting PTSD or PTSS among individuals with diabetes and reporting prevalence estimates and/or associated factors from the inception to January 1, 2026. Study characteristics, outcome measures, prevalence estimates, associated factors, and reported trauma sources were charted. PTSD, PTSS, medical traumatic stress, and diabetes distress were treated as distinct constructs. A detailed protocol for this scoping review was registered in the Open Science Framework (OSF) prior to the initiation of literature search (Registration doi:10.17605/OSF.IO/DMVNH).

**Results:**

A total of 11 reports representing 10 independent study populations were included, of which 10 adopted a cross‐sectional design and one retrospective registry‐based study. The studies involved diverse diabetes populations and used heterogeneous PTSD/PTSS assessment approaches. Reported prevalence estimates ranged from 0.12% to > 50%, reflecting substantial heterogeneity in study populations, diabetes characteristics, outcome definitions, assessment instruments, and settings. Reported associated factors included demographic and clinical characteristics, diabetes‐related experiences and complications, psychological distress, and diabetes self‐management characteristics. Some studies examined diabetes‐related medical experiences as potential trauma sources, whereas others reported nondiabetes‐related trauma or did not specify the trauma source. Associations with glycemic control, self‐management, psychological distress, and quality of life were reported in some studies; however, the predominantly cross‐sectional evidence limits causal interpretation.

**Conclusions:**

PTSD/PTSS have been reported among individuals with diabetes, but the available estimates are highly heterogeneous and should not be interpreted as a pooled or directly comparable prevalence. Future research should use clearer and more consistent definitions of PTSD/PTSS and trauma exposure, distinguish formal PTSD diagnoses from symptom‐based outcomes, and use longitudinal designs to clarify their temporal and clinical relationships with diabetes.

## 1. Introduction

Diabetes mellitus (DM) is one of the most prevalent chronic noncommunicable diseases worldwide and represents a major public health challenge because of its increasing prevalence, lifelong management requirements, and substantial healthcare costs [1]. According to the International Diabetes Federation (IDF) Diabetes Atlas (11th edition), approximately 589 million adults worldwide were living with diabetes in 2025, representing nearly one in nine adults globally [2]. Beyond metabolic and vascular complications, diabetes requires continuous self‐management, including glucose monitoring, medication use, dietary regulation, and adaptation to treatment demands, which may impose substantial psychological burden [3, 4]. Although depression, anxiety, and diabetes distress are well‐recognized psychological concerns in diabetes, increasing evidence suggests that some individuals may also experience posttraumatic stress disorder (PTSD) or posttraumatic stress symptoms (PTSS) in relation to diabetes‐related or other traumatic experiences [5, 6].

PTSD is a trauma‐ and stressor‐related disorder characterized by persistent re‐experiencing, avoidance, alterations in cognition and mood, and hyperarousal following exposure to a qualifying traumatic event [7, 8]. PTSS refers to PTSS that do not necessarily fulfill all diagnostic criteria for PTSD and should not be interpreted as equivalent to a formal PTSD diagnosis. Similarly, diabetes distress describes the emotional burdens and worries associated with living with and managing diabetes and is conceptually distinct from PTSD and depression [9]. Diabetes‐related experiences such as severe hypoglycemia, diabetic ketoacidosis (DKA), emergency hospitalization, and intensive medical interventions may be perceived as traumatic by some individuals and have been examined as potential sources of PTSS [3, 10]. However, medical severity alone does not establish DSM‐5‐TR Criterion A trauma [11]. Routine self‐management, fear of complications, and treatment burden may cause substantial diabetes distress without necessarily constituting traumatic exposure [9]. Accordingly, interpretation of PTSD/PTSS in diabetes requires consideration of both the outcome definition and the trauma referent.

Existing studies have reported associations between PTSD/PTSS and diabetes‐related outcomes. Clinically documented PTSD has been associated with poorer glycemic control, hospitalization, and DKA among individuals with Type 1 diabetes [12]. Diabetes‐related PTSS has also been associated with depression, anxiety, and diabetes distress among adolescents and young adults with Type 1 diabetes [3]. Studies in adults with Type 2 diabetes have further reported associations between PTSD severity and diabetes‐related complications and other clinical outcomes [13, 14]. However, the available evidence is heterogeneous in trauma referents, diabetes populations, assessment instruments, diagnostic thresholds, and outcome definitions. Some studies focus on diabetes‐related acute medical events, whereas others examine broader medical traumatic stress, combined diabetes‐ and nondiabetes‐related trauma, or general PTSD/PTSS without a clear diabetes‐specific trauma referent. Most studies are also observational, limiting causal interpretation.

Therefore, this scoping review is aimed at mapping the available evidence on PTSD/PTSS among individuals with diabetes, with particular attention to the conceptualization and assessment of diabetes‐related trauma. Specifically, we sought to describe the characteristics of the available studies, identify the trauma referents and diabetes‐related experiences examined, summarize reported PTSD/PTSS prevalence or severity and assessment approaches, and synthesize reported correlates. By distinguishing diabetes‐related trauma from general trauma, medical traumatic stress, and diabetes distress, this review is aimed at providing a clearer framework for interpreting the heterogeneous evidence in this emerging field.

## 2. Methodology

This scoping review was conducted in accordance with the Arksey and O′Malley framework, which is the most widely used methodological guideline for scoping reviews, supplemented by the recommendations from the Joanna Briggs Institute (JBI) Scoping Review Manual to ensure methodological rigor [15, 16]. The framework consists of five key stages: (1) identifying the research question, (2) identifying relevant studies, (3) selecting studies, (4) charting the data, and (5) collating, summarizing, and reporting the results. A detailed protocol for this scoping review was registered in the Open Science Framework (OSF) prior to the initiation of literature search. (Registration doi:10.17605/OSF.IO/DMVNH). The registered protocol initially focused on PTSD; during the review process, the scope was expanded to include PTSS because a substantial proportion of the identified studies assessed PTSD‐related symptoms using validated symptom‐based instruments rather than formal diagnostic procedures. This amendment was made to capture the full range of PTSD‐related evidence and to distinguish symptom‐based outcomes from formal PTSD diagnoses. The amendment and its rationale are reported transparently in this review.

### 2.1. Research Question

Guided by the Population, Concept, and Context (PCC) framework, commonly adapted for scoping reviews to refine the research scope [16]. This review sought to map the available evidence on the reported prevalence of PTSD and PTSS, and factors reported in association with these outcomes among individuals with diabetes. The review is also aimed at characterizing the reported source or referent of trauma and distinguishing PTSD/PTSS from related but conceptually distinct constructs, including medical traumatic stress and diabetes distress. Specifically, the PCC components were defined as follows:

• Population: Individuals of any age, sex, or ethnic background diagnosed with any subtype of diabetes, including Type 1 diabetes, Type 2 diabetes, gestational diabetes, and other specified forms of diabetes.
• Concept: PTSD and/or PTSS identified through clinical diagnostic criteria, clinician‐administered interviews, diagnostic codes, or validated PTSD/PTSS assessment instruments. PTSD/PTSS were considered according to the outcome definition reported in each study and were not assumed to represent equivalent diagnostic constructs.
• Context: Studies conducted in any geographic region or healthcare or community setting, including primary care, hospitals, specialty clinics, and community settings, and using quantitative, qualitative, or mixed‐methods designs.

### 2.2. Identifying Relevant Studies

Two researchers (Z.Y. and C.R.) independently searched Web of Science, PubMed, Embase, Cochrane Library, CNKI, Wan Fang Data, and VIP databases through the computer system. The search period was from the inception to January 1, 2026. To ensure comprehensive search, the review included studies examining PTSD, PTSS, and related constructs among individuals with diabetes.

Search terms were developed using combinations of terms related to diabetes and PTSD/PTSS. The PubMed search strategy is presented in Table 1, and equivalent strategies adapted to the syntax and indexing systems of the other databases are provided in Table S1.

**Table 1:** Search strings in this study from PubMed.

Item	Search strategy	Results
#1 Population	“Diabetes Mellitus”[Mesh] OR “Type 1 Diabetes”[Title/Abstract] OR “Type 2 Diabetes”[Title/Abstract] OR “Diabetic Patients”[Title/Abstract] OR “Diabetes”[Title/Abstract]	921,877
#2 Concept/outcome	“Stress Disorders, Post‐Traumatic”[Mesh] OR “PTSD”[Title/Abstract] OR “post traumatic stress disorder”[Title/Abstract] OR “posttraumatic stress disorder”[Title/Abstract] OR “PTSS”[Title/Abstract] OR “post traumatic stress symptom∗”[Title/Abstract] OR “posttraumatic stress symptom∗”[Title/Abstract] OR “traumatic stress”[Title/Abstract] OR “medical traumatic stress”[Title/Abstract]	69,966
#3	#1 AND #2	378

### 2.3. Eligibility Criteria

Studies were eligible if they met the following criteria: (1) study population: included individuals diagnosed with any subtype diabetes of any age, gender, or ethnic background; (2) concept: Studies that assessed PTSD and/or PTSS using clinical diagnostic criteria, structured diagnostic interviews, or validated PTSD/PTSS assessment instruments; (3) trauma referent: The study reported, assessed, or allowed identification of the trauma referent where available. Trauma sources were subsequently classified as diabetes‐related, nondiabetes‐related, mixed, or unclear during data charting. (4) Outcome measures: studies reporting the prevalence, frequency, severity, or distribution of PTSD/PTSS, or reporting factors statistically or qualitatively associated with PTSD/PTSS; (5) study design: any empirical study design, including cross‐sectional studies, cohort studies, case‐control studies, qualitative studies, mixed‐methods studies, and observational studies; (6) publication type: full‐text peer‐reviewed journal articles.

Studies were excluded if they met any of the following criteria: (1) did not include individuals with diabetes; (2) did not assess a PTSD/PTSS‐specific outcome, including studies assessing only depression, anxiety, diabetes distress, or general psychological distress without a PTSD/PTSS measure; (3) review articles such as systematic reviews, meta‐analyses and narrative reviews, editorial comments, letters to the editor, or commentaries without original empirical data; (4) studies with abstract‐only publications without full‐text availability; (5) did not provide sufficient information to determine that PTSD/PTSS was assessed in the diabetes population.

### 2.4. Study Selection

All records retrieved from the included databases were imported into NoteExpress for reference management and deduplication. Duplicate records were identified using the software′s duplicate‐detection function and subsequently checked manually to verify and remove residual duplicates.

The study selection process was conducted independently by two researchers (Z.Y. and C.R.) in two consecutive stages: title and abstract screening, followed by full‐text screening. Disagreements between the two researchers at any stage were resolved through discussion and consensus; if consensus could not be reached, a third independent researcher (J.Y.) was consulted to make the final decision. A PRISMA‐ScR (Preferred Reporting Items for Systematic Reviews and Meta‐Analyses Extension for Scoping Reviews) flow diagram was used to document the study selection process (Table S2), including the number of studies identified, excluded, and included at each stage [17].

### 2.5. Data Charting

The research team developed a standardized data extraction form through discussion, following the JBI scoping review methodology [16]. The data charting form included the following key items: (1) study characteristics: first author, year of publication, country/region, study design, sample size, age and gender distribution of the study population, and diabetes subtype; (2) assessment tools: Instruments used to diagnose or assess PTSD, such as PTSD checklist for DSM‐5 [PCL‐5], Clinician‐Administered PTSD Scale (CAPS/CAPS‐IV), and Impact of Event Scale‐Revised (IES‐R); (3) prevalence data: prevalence or frequency of PTSD/PTSS, sample denominator, subgroup estimates, symptom severity, and relevant confidence intervals or other uncertainty estimates where reported; (4) trauma referent: index traumatic event or trauma source, including diabetes‐related, nondiabetes‐related, mixed, or unclear/not reported; (5) reported correlates: demographic, clinical, diabetes‐related, psychological, social, and trauma‐related factors reported in statistical or qualitative association with PTSD/PTSS, together with effect estimates, confidence intervals, and *p* values where available.

Data extraction was conducted independently by two researchers (Z.Y. and C.R.), with each researcher extracting data from half of the included studies and cross‐checking the other half to minimize errors. Any discrepancies in data extraction were resolved through discussion between the two researchers; if consensus was not achieved, a third researcher (J.Y.) was consulted. Missing data from included studies were not imputed; instead, the study was noted as having incomplete data for the relevant item, and attempts were made to contact the study authors for additional information where feasible.

### 2.6. Data Synthesis and Reporting

Consistent with the objectives of scoping reviews, a narrative synthesis approach was used to collate, summarize, and report the results, rather than a meta‐analysis due to the expected heterogeneity in study populations, assessment tools, and methodologies [16]. The synthesis was structured around the two core objectives of the review: (1) reported prevalence and characteristics of PTSD/PTSS among individuals with diabetes; and (2) factors reported in association with PTSD/PTSS. For prevalence estimates, findings were summarized according to diabetes subtype, age group, trauma referent, assessment approach, and outcome definition. Reported correlates were categorized according to demographic, clinical, diabetes‐related, psychological, social, and trauma‐related characteristics, with adjusted and unadjusted associations distinguished where reported.

Given the predominantly observational and cross‐sectional nature of the evidence, reported associations were interpreted as associated factors or correlates rather than causal risk factors.

### 2.7. Ethical Considerations

This scoping review is based solely on previously published articles and does not involve any original research involving humans or animals; therefore, ethical approval is not required.

## 3. Results

The initial search identified 2511 records, of which 1428 duplicate records were removed, leaving 1083 records for title and abstract screening. Following screening, 863 records were excluded, and 220 reports were sought for full‐text retrieval. Of these, 27 reports could not be retrieved, leaving 193 reports for full‐text eligibility assessment. Following full‐text assessment, 182 reports were excluded because the participants did not meet the eligibility criteria (*n* = 137), the outcomes did not meet the eligibility criteria (*n* = 44), or relevant data were unavailable (*n* = 1). Ultimately, 11 reports representing 10 independent study populations met the eligibility criteria and were included in the review. The study selection process is presented in Figure 1.

**Figure 1:**
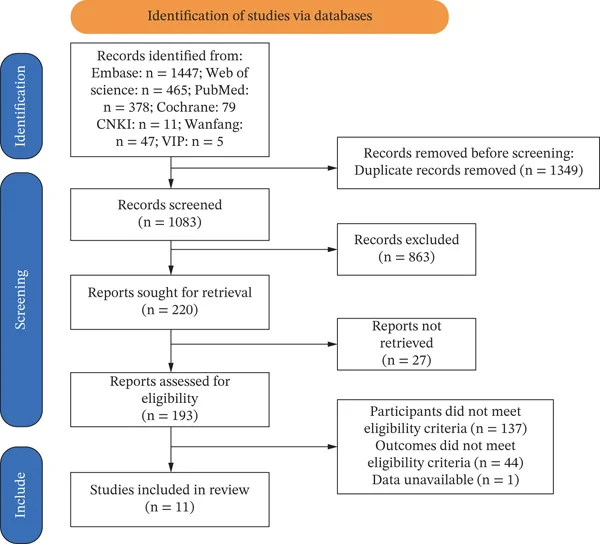
Flow diagram of the study selection.

### 3.1. Characteristics of Included Studies

Eleven reports representing 10 independent study populations were included in the review, two reports by Hosoda‐Urban and O′Donnell [3, 10] were derived from the same underlying U.S. cohort of 50 adolescents and young adults with Type 1 diabetes and were therefore treated as multiple reports from a single study rather than independent studies. The studies were published between 2012 and 2025 and were conducted across multiple regions, including Asia (China and Turkey), Europe Germany, Austria, Switzerland, and Luxembourg), and North America (United States). In terms of study design, 10 studies were cross‐sectional, and one study employed a retrospective registry‐based design. Sample sizes varied considerably, ranging from 40 to 152,265 participants. The characteristics of study populations were heterogeneous, including children, adolescents, young adults, and adults. Several studies focused specifically on individuals with Type 1 diabetes, particularly in pediatric and adolescent populations, whereas others included participants with Type 2 diabetes or mixed diabetes subtypes. The studies were conducted in a variety of settings, including outpatient diabetes clinics, hospital‐based services, community‐based samples, and population‐based registries. Detailed characteristics of the included studies are presented in Table 2.

**Table 2:** Characteristics and main findings of the included reports.

Author (year)	Country/region	Study design	Diabetes type (*n*)	PTSD assessment/diagnostic instrument	PTSD prevalence/severity	Trauma referent/index event	Diabetes‐related trauma
Li et al. [18] (2025)	China	Cross‐sectional	Type 2 (242)	Impact of Event Scale–Revised (IES‐R)	PTSD prevalence 10.33% (IES‐R ≥ 33); total score range 0–88	COVID pandemic‐related PTSD symptoms	No/not diabetes‐specific
Hosoda‐Urban et al. [3] (2025)	United States	Cross‐sectional	Type 1 (50)	Adults: PTSD Diagnostic Scale for DSM‐5 (PDS‐5); adolescents: Child PTSD Symptom Scale for DSM‐5 (CPSS‐5‐SR)	Clinically relevant PTSS: 28.0% (mild 18.0%, moderate 8.0%, severe 2.0%)	Diabetes‐related events/medical trauma	Yes
Hosoda‐Urban et al. [10] (2024)	United States	Cross‐sectional	Type 1 (50)	PDS‐5 (adults); CPSS‐5‐SR (adolescents)	Clinically relevant PTSS: 28.0%; mean score 8.80 (SD 10.80)	General trauma screened first; most distressing event subsequently classified as diabetes‐related or nondiabetes‐related	Yes, explicitly anchored
Raj et al. [19] (2022)	United States	Cross‐sectional	Type 1 (99)	Child and Adolescent Trauma Screen (CATS)	Trauma exposure: 65.7%; PTSD positive screening: 38.4% (including symptoms and functional impairment)	Accidental injury (28.3%), medical traumatic stress (22.2%), natural disaster (10.1%), witnessing family violence (6.1%)	Unclear; not specifically diabetes‐anchored
Powers et al. [20] (2016)	United States	Cross‐sectional	Type 2 (40)	Clinician‐Administered PTSD Scale (CAPS/CAPS‐IV)	Current PTSD prevalence 32.5% (*n* = 13)	Lifetime trauma exposure and childhood abuse	No; not diabetes‐specific
Lunkenheimer et al. [12] (2023)	Germany, Austria, Switzerland, Luxembourg	Retrospective	Type 1 (152,265)	ICD‐10 (F43.1)/DSM‐5 (309.81) diagnostic codes	Clinically diagnosed PTSD prevalence 0.12% (*n* = 179)	No specific index trauma available from registry data	Unclear/not assessed
Renna et al. [21] (2016)	United States	Cross‐sectional	Type 1 (239)	PTSD Diagnostic Scale (PDS)	Standard PTSD score: 31%; conservative PTSD score: 10%; mean PDS severity score: 10.6	Hyperglycemia‐related events; hypoglycemia and perceived death threat also assessed	Yes
Lunkenheimer et al. [22] (2023)	Germany	Cross‐sectional	Type 1 (177,T1D subgroup)	Child and Adolescent Trauma Screen (CATS)	Self‐reported clinical PTSS (CATS ≥ 21): 35.7%; observer‐reported: 47.1%	Psychosocial factors (anxiety, depression, coping style, personal growth, general health); diabetes‐related stressful events	Yes, but not diabetes‐specific
Şişmanlar et al. [23] (2012)	Turkey	Cross‐sectional	Type 1 (42)	Children′s PTSD Reaction Index (CPTS‐RI)	Moderate‐to‐severe PTSS: 51.9%; severe‐to‐extreme PTSS: 18.5%	Hypoglycemia as potential traumatic event	Yes
Aronson et al. [24] (2016)	United States (American Indian communities)	Cross‐sectional	Type 2 (218)	Primary Care PTSD Screen (PC‐PTSD)	Positive PTSD screening (score ≥ 3): 21.8%	Lifetime/general traumatic experiences; diabetes outcomes examined separately	No/not specifically diabetes‐anchored
Dixon et al. [25] (2020)	United States (Atlanta)	Cross‐sectional	Type 1 and Type 2 (290, [91.4% Type 2])	Modified PTSD Symptom Scale (MPSS); CAPS‐IV/CAPS‐5	Current PTSD prevalence 30.4%; higher in Type 2 diabetes subgroup (*n* = 96)	Childhood and nonchildhood abuse trauma	No; not diabetes‐specific

### 3.2. PTSD Assessment and Diagnostic Instruments

The included studies used heterogeneous approaches to assess PTSD, including clinician‐administered diagnostic interviews, registry‐based diagnostic codes, self‐report screening instruments, and symptom severity measures. Clinician‐administered assessments included the CAPS/CAPS‐IV, whereas one large retrospective registry study identified PTSD using ICD‐10 and DSM‐5 diagnostic codes. Self‐report instruments included the Impact of Event Scale–Revised (IES‐R), PTSD Diagnostic Scale (PDS/PDS‐5), Child PTSD Symptom Scale for DSM‐5 (CPSS‐5), Child and Adolescent Trauma Screen (CATS), Child PTSD Symptom Reaction Index (CPTS‐RI), Primary Care PTSD Screen (PC‐PTSD), and Modified PTSD Symptom Scale (MPSS).

Two studies employed clinician‐administered diagnostic interviews, including the Clinician‐Administered PTSD Scale (CAPS/CAPS‐IV) [20, 25]. One large retrospective study identified PTSD cases using ICD‐10 (F43.1) and DSM‐V (309.81) diagnostic codes [21]. PTSD outcomes were reported either as categorical diagnoses, screening positivity, or symptom severity scores, with variable cutoff thresholds (e.g., IES‐R ≥ 33, CATS ≥ 21, PC‐PTSD ≥ 3). The specification of an index traumatic event and its relationship to diabetes varied across studies, and assessment of DSM‐5 Criterion A was inconsistently reported. No diabetes‐specific PTSD assessment instrument was identified.

### 3.3. PTSD/PTSS Prevalence Estimates

Reported PTSD/PTSS prevalence estimates varied across studies according to population, study design, assessment instrument, and case definition. Therefore, estimates were summarized descriptively rather than directly compared. Among adults with Type 2 diabetes, reported PTSD/PTSS estimates ranged from 10.33% based on an IES‐R threshold to 32.5% based on clinician‐administered CAPS/CAPS‐IV assessment. These estimates should not be interpreted as directly comparable because they represented different outcome definitions and assessment approaches. A large retrospective European registry study identified PTSD in 0.12% (179/152,265) of individuals with Type 1 diabetes based on recorded ICD‐10/DSM diagnostic codes [21].

Among individuals with Type 1 diabetes, particularly adolescents and young adults, higher rates of PTSS were observed. One U.S. cohort of adolescents and young adults with Type 1 diabetes reported clinically relevant PTSS in 28.0% of participants [10]; the same cohort was reported in two publications and was therefore not treated as independent prevalence evidence. Another adolescent study reported a trauma exposure rate of 65.7%, with 38.4% screening positive for PTSD, defined by both symptom endorsement and functional impairment [19]. Additional studies reported CATS‐based PTSS prevalence of 35.7% (self‐report) and 47.1% (observer report) [23], as well as moderate‐to‐severe PTSS in 51.9% of participants [24].

In mixed or special populations, PTSD screening positivity ranged from 21.8% (PC‐PTSD ≥ 3) to 30.4%, with one study reporting higher PTSD prevalence in the Type 2 diabetes subgroup (35.4%, 34/96) compared with those with Type 1 diabetes.

### 3.4. Reported Correlates of PTSD/PTSS

Several demographic, psychological, clinical, and diabetes‐related characteristics were reported in association with PTSD/PTSS. Female sex, greater diabetes‐related distress or hardship, and higher depressive and anxiety symptoms were associated with greater PTSD/PTSS burden. Other reported correlates included emotion dysregulation, coping and resilience, difficulties with diabetes self‐management, and distress related to diabetes‐related events. Diabetes‐related experiences, including hypoglycemia, hyperglycemia‐related events, diabetes complications, and diabetes‐related emergency or medical care, were also reported in relation to PTSD/PTSS.

Nondiabetes‐related trauma exposures, including childhood maltreatment, abuse, accidental injury, family violence, natural disasters, and other general traumatic experiences, were also reported among individuals with diabetes. Given the predominance of cross‐sectional designs and substantial heterogeneity in outcome definitions and analytical approaches, these findings represent reported correlates rather than causal risk factors.

## 4. Discussion

### 4.1. Overview of Principal Findings and Conceptualization of Trauma

This scoping review identified substantial heterogeneity in the conceptualization of trauma among individuals with diabetes. Some studies explicitly linked PTSS to diabetes‐related experiences, including hyperglycemia, hypoglycemia, severe diabetes‐related symptoms, complications, and emergency care [3, 19, 23]. In contrast, other studies examined PTSD following childhood maltreatment, abuse, general trauma, or other external stressors among people with diabetes [20, 24, 25]. Thus, the available evidence does not support the assumption that PTSD in diabetes is predominantly attributable to illness‐related trauma.

Importantly, diabetes distress, chronic disease burden, and perceived loss of control should not automatically be equated with PTSD‐related trauma. Under DSM‐5/DSM‐5‐TR, PTSD requires exposure to actual or threatened death, serious injury, or sexual violence, and therefore not all distressing diabetes experiences necessarily meet the trauma criterion (American Psychiatric Association, 2013; 2022). The findings suggest that some diabetes‐related events may represent clinically relevant traumatic stressors, but clearer distinction between diabetes‐related trauma, medical traumatic stress, diabetes distress, and PTSD/PTSS is needed in future research.

### 4.2. Measurement Heterogeneity and Interpretation of PTSD/PTSS Estimates

Measurement heterogeneity was a major challenge in interpreting PTSD/PTSS estimates across the included studies. The 11 reports used self‐report instruments, clinician‐administered interviews, and routinely recorded diagnostic codes, with different thresholds and outcome definitions. For example, Powers et al. reported current PTSD in 32.5% of 40 adults with Type 2 diabetes using the CAPS/CAPS‐IV, whereas Lunkenheimer et al. identified documented PTSD in only 0.12% of 152,265 individuals with type 1 diabetes using registry diagnoses [20, 22]. These outcomes are not directly equivalent because clinical diagnosis and symptom screening capture different levels of diagnostic certainty.

Similarly, symptom‐based studies reported 28.0% clinically relevant diabetes‐related PTSS among 50 adolescents and young adults with Type 1 diabetes using the PDS‐5/CPSS‐5‐SR [19], 51.9% moderate‐or‐higher PTSS among children with Type 1 diabetes using the CPTS‐RI [23], and more than 30% hyperglycemia‐related PTS using the PDS in 239 adults with Type 1 diabetes [3]. These differences may reflect variation in age, diabetes type, sample size, trauma referent, instrument, and diagnostic threshold rather than differences in the underlying prevalence of PTSD. Therefore, prevalence estimates across the included studies should be interpreted descriptively rather than compared as equivalent measures of PTSD burden. Standardized assessment and explicit identification of the index traumatic event are needed to improve comparability across future studies.

### 4.3. Reported Correlates and Potentially Protective Factors

The included studies reported heterogeneous correlates of PTSD/PTSS across demographic, psychological, and diabetes‐related domains. Female sex, greater diabetes‐related hardship or distress, and higher depressive or anxiety symptoms were associated with greater PTSD/PTSS burden in some studies [5, 19]. Diabetes self‐management difficulties and fear of hypoglycemia were also associated with greater posttraumatic stress symptoms, whereas resilience and adaptive coping were associated with more favorable psychological outcomes [19]. Other studies linked PTSD to prior trauma exposure, childhood maltreatment, and emotion regulation difficulties [20, 25].

These findings should be interpreted cautiously. Depression, anxiety, and diabetes distress may represent co‐occurring or overlapping psychological constructs rather than causal determinants of PTSD/PTSS. Moreover, the reported associations were derived mainly from cross‐sectional studies, and the available evidence did not consistently distinguish adjusted associations from descriptive or unadjusted findings. Accordingly, these variables are best regarded as reported correlates or potentially protective characteristics rather than established risk or protective factors.

### 4.4. Clinical and Research Implications

The findings suggest that psychological assessment in diabetes may benefit from distinguishing PTSD/PTSS from diabetes distress and other forms of illness‐related psychological burden. In particular, assessment should clarify the presence and nature of the index traumatic event rather than assuming that diabetes itself constitutes a traumatic exposure. Current diabetes care guidance supports routine psychosocial assessment and referral for concerns such as diabetes distress, depression, and anxiety, providing a relevant framework for broader psychological assessment in diabetes care [5, 8].

Future research should use clearly defined trauma referents and standardized PTSD diagnostic or assessment instruments, while distinguishing formal PTSD from PTSS, medical traumatic stress, and diabetes distress. Longitudinal studies with adequately powered samples are also needed to clarify temporal relationships between diabetes‐related experiences and PTSD/PTSS. Intervention studies are warranted before specific trauma‐informed approaches can be recommended for diabetes care.

### 4.5. Strengths and Limitations

This review has several strengths. It systematically mapped the available evidence on PTSD/PTSS in people with diabetes while specifically examining trauma referents and the extent to which traumatic experiences were diabetes‐related. The inclusion of different diabetes types, age groups, settings, and assessment approaches also allowed methodological and conceptual heterogeneity to be identified.

Several limitations should be considered. First, only 11 reports met the inclusion criteria, and most were cross‐sectional, limiting conclusions regarding temporal or causal relationships. Second, substantial heterogeneity existed in trauma definitions, study populations, PTSD/PTSS instruments, diagnostic thresholds, and outcome definitions, precluding direct comparison or quantitative synthesis of prevalence estimates. Third, the evidence was concentrated mainly in North America and Europe, limiting its generalizability to other populations and healthcare contexts. Finally, some reports may represent overlapping samples, which should be considered when interpreting the apparent breadth of the evidence base.

## 5. Conclusion

This scoping review identified limited and heterogeneous evidence on PTSD/PTSS among individuals with diabetes, with only a subset of studies explicitly examining diabetes‐related trauma. Differences in trauma referents, populations, assessment instruments, and diagnostic thresholds substantially limit direct comparison of reported estimates. Reported correlates, including psychological and diabetes‐related factors, should be interpreted as associations rather than causal risk factors. Future research should clearly define the index traumatic event, distinguish PTSD/PTSS from diabetes distress and medical traumatic stress, and use standardized and longitudinal approaches to clarify the nature and burden of diabetes‐related traumatic stress.

## Author Contributions

Zhang Yuanping and Cao Ruiqing: design of the study, development of the search strategy, and preparation of the first draft. Zhang Yuanping, Cao Ruiqing, Ke Xiong, and Jing Yuanyuan: database searching, study selection, data extraction, analysis, and synthesis, critical review, and revision of the manuscript. Jing Yuanyuan: study supervision.

## Funding

This study was supported by the 2025 Project of Nanchong Social Science Research “14th Five‐Year Plan” Period, Nanchong Federation of Social Sciences Circles (NC25B209).

## Disclosure

All authors reviewed the manuscript and approved the final version.

## Ethics Statement

The authors have nothing to report.

## Conflicts of Interest

The authors declare no conflicts of interest.

## Supporting information


**Supporting Information 1** Additional supporting information can be found online in the Supporting Information section. Table S1: The equivalent strategies adapted to the syntax and indexing systems of the other database. Table S2: PRISMA‐ScR checklist for the present scoping review.

## Data Availability

No datasets were generated or analyzed during the current study.
